# Pulmonary Effects of Indoor- and Outdoor-Generated Particles in Children with Asthma

**DOI:** 10.1289/ehp.7511

**Published:** 2005-01-10

**Authors:** Jane Q. Koenig, Therese F. Mar, Ryan W. Allen, Karen Jansen, Thomas Lumley, Jeffrey H. Sullivan, Carol A. Trenga, Timothy V. Larson, L.-Jane S. Liu

**Affiliations:** ^1^Department of Environmental Health and Occupational Sciences,; ^2^Department of Biostatistics, and; ^3^Department of Civil and Environmental Engineering, University of Washington, Seattle, Washington, USA

**Keywords:** ambient air pollution, asthma, exhaled nitric oxide, infiltration, PM_2.5_

## Abstract

Most particulate matter (PM) health effects studies use outdoor (ambient) PM as a surrogate for personal exposure. However, people spend most of their time indoors exposed to a combination of indoor-generated particles and ambient particles that have infiltrated. Thus, it is important to investigate the differential health effects of indoor- and ambient-generated particles. We combined our recently adapted recursive model and a predictive model for estimating infiltration efficiency to separate personal exposure (*E*) to PM_2.5_ (PM with aerodynamic diameter ≤2.5 μm) into its indoor-generated (*E*_ig_) and ambient-generated (*E*_ag_) components for 19 children with asthma. We then compared *E*_ig_ and *E*_ag_ to changes in exhaled nitric oxide (eNO), a marker of airway inflammation. Based on the recursive model with a sample size of eight children, *E*_ag_ was marginally associated with increases in eNO [5.6 ppb per 10-μg/m^3^ increase in PM_2.5_; 95% confidence interval (CI), −0.6 to 11.9; *p* = 0.08]. *E*_ig_ was not associated with eNO (−0.19 ppb change per 10μg/m^3^). Our predictive model allowed us to estimate *E*_ag_ and *E*_ig_ for all 19 children. For those combined estimates, only *E*_ag_ was significantly associated with an increase in eNO (*E*_ag_: 5.0 ppb per 10-μg/m^3^ increase in PM_2.5;_ 95% CI, 0.3 to 9.7; *p* = 0.04; *E*_ig_: 3.3 ppb per 10-μg/m^3^ increase in PM_2.5_; 95% CI, −1.1 to 7.7; *p* = 0.15). Effects were seen only in children who were not using corticosteroid therapy. We conclude that the ambient-generated component of PM_2.5_ exposure is consistently associated with increases in eNO and the indoor-generated component is less strongly associated with eNO.

It is known that particulate matter (PM) air pollution is associated with both increased morbidity and mortality [Brunekreef 1997; Koenig 2000; Pope 2000; Sunyer 2001; U.S. Environmental Protection Agency (EPA) 2004]. In many residences, ambient fine particles readily penetrate indoors (Abt et al. 2000; Allen et al. 2003; Anuszewski et al. 1998; Long et al. 2001; Sarnat et al. 2002), where most people spend > 90% of their time. As a result, individuals receive a substantial fraction of their exposure to ambient-generated particles while they are indoors. Therefore, it is important to evaluate the differential health effect of particles generated outdoors from those generated indoors. This information is needed both for health risk estimates and regulatory control to protect public health.

Most health effects studies have tested for associations between measures of ambient PM and adverse health effects. Only a few studies have evaluated the relative toxicity of indoor versus outdoor PM. One study assessed the *in vitro* toxicity of paired indoor and outdoor PM_2.5_ (PM with aerodynamic diameter ≤2.5 μm) samples collected in homes in Boston, Massachusetts (Long et al. 2001). The *in vitro* test used rat alveolar macrophages and measured change in tumor necrosis factor α(TNF-α) as a marker for inflammation. PM_2.5_ from both outdoor and indoor samples increased endotoxin-normalized TNF-α levels significantly; however, the increases were greater for indoor PM samples (mean, 952 ± 157 pg/endotoxin unit vs. 494 ± 96 pg/endotoxin unit).

Another study evaluated the influence of air conditioning on observed associations between outdoor PM and health outcomes (Janssen et al. 2002). Health data for hospital admissions for chronic obstructive pulmonary disease (COPD) and cardiovascular disease were obtained for 14 U.S. cities. Home air conditioning was associated with lower penetration of outdoor particles, and the associations between PM_10_ and hospital admissions were lower in cities with a higher prevalence of air conditioning.

In a recent panel study of 16 subjects with COPD in Vancouver, Canada, Ebelt et al. (in press) developed separate estimates of exposures to ambient and nonambient (i.e., the sum of indoor-generated particles and particles generated from personal activities) particles of different size ranges (PM_2.5_, PM_10–2.5_, and PM_10_) based on time–activity data and the use of particle sulfate measurements as a tracer of ambient particles. Health outcomes were examined against these estimated exposures. Total and nonambient particle exposures were not associated with any of the health outcomes, whereas estimated ambient exposures and, to a lesser extent, ambient concentrations were associated with decreased lung function, decreased systolic blood pressure, increased heart rate, and increased supraventricular ectopic heart beats.

We recently described a technique for separating personal exposure to PM into its indoor- and ambient-generated components using hourly light scattering data and a recursive modeling technique (Allen et al. 2003). The data came from a large panel study in Seattle, Washington, that collected indoor, outdoor, and personal exposure data on 107 subjects over a 2-year period (Liu et al. 2003). The Seattle study also collected various health end points that included lung function and exhaled nitric oxide (eNO), a marker of airway inflammation, in a subset of children with asthma. In a previous article we reported eNO associations with 24-hr PM_2.5_ concentrations measured outside the home [4.3 ppb increase in eNO per 10-μg/m^3^ increase in PM_2.5_; 95% confidence interval (CI), 1.4 to 7.2], inside the home (4.2 ppb; 95% CI, 1.0 to 7.4), and on subjects (4.5 ppb; 95% CI, 1.0 to 7.9) (Koenig et al. 2003). In this article we describe the results of analyzing further the health data to test the associations between health outcomes and estimates of indoor-generated exposure (*E*_ig_) and ambient-generated exposure (*E*_ag_) based on subject time–location data and estimated particle infiltration efficiency (*F*_inf_; the fraction of the outdoor concentration that penetrates indoors and remains suspended). We hypothesize that PM_2.5_ of outdoor origin has more effect on respiratory outcomes per unit mass than particles of indoor origin.

## Materials and Methods

This study was conducted between winter 2000–2001 and spring 2001 in Seattle, Washington, as part of a larger exposure assessment and health effect panel study (Liu et al. 2003). Nineteen children, 6–13 years of age, were recruited from a local asthma and allergy clinic. All had physician-diagnosed asthma and were prescribed asthma medications daily or regularly. Ten of the subjects were not using inhaled corticosteroid (ICS) medication; nine were. Each subject in the panel was asked to participate for a 10-day monitoring session. Trained technicians made daily home visits to subjects between 1700 and 2000 hr to take air and health effect measurements.

### Pollutant concentration measurements.

PM measurements were taken inside and outside of each subject’s residence using the Harvard impactors for integrated PM_2.5_ (HI_2.5_) concentrations and using the Radiance nephelometer (model 903; Radiance Research, Seattle, WA) at eight residences for continuous light-scattering measurements. Personal PM_2.5_ measurements were collected from each subject using the Harvard personal environmental monitors. Detailed descriptions and evaluation of these samplers can be found in Liu et al. (2002). All integrated measurements were collected over 24 hr (~ 1600 to 1600 hr) for 10 consecutive days. In addition, NO concentrations were monitored continuously at the Beacon Hill central site using a chemiluminescence monitor operated by the Washington State Department of Ecology (Olympia, WA).

### Measurement of NO.

Exhaled breath measurements were collected offline daily in the children’s homes into an NO inert and impermeable Mylar balloon for up to 10 consecutive days. Samples were collected in the afternoon or early evening at the child’s residence. Children were asked to forgo food intake for 1 hr before collection of exhaled breath. Exhaled breath was collected before lung function measurements, because deep inspirations affect NO concentration (Deykin et al. 1998). NO was quantified within 24 hr of collection using an API (Advanced Pollution Instrumentation, Inc., San Diego, CA) chemiluminescent nitrogen oxides (NO_x_) monitor (model 200A). We have tested the stability of NO in the Mylar bags by running comparisons of values immediately after collection and at 24 and 48 hr after collection and found NO values varying by < 2 ppb (*n* = 8). A complete description of the methods has been published (Koenig et al. 2003).

### Measurement of lung function.

During the daily visits, coached spirometry values consistent with American Thoracic Society criteria (American Thoracic Society 1995) were obtained with MicroDL spirometers (Micro Medical, Lewiston, ME). Spirometry measurements included forced expiratory volume in 1 sec (FEV_1_), forced vital capacity (FVC), and mid-expiratory flow (MEF). In addition, symptom forms were completed by subjects and medication use during the previous 24 hr was reviewed and collected. Subjects also filled out a time–location–activity diary (TAD) with a 15-min resolution.

### Estimation of PM exposure components.

We previously described the use of a recursive mass balance model (RM) to estimate the average *F*_inf_ for individual residences (Allen et al. 2003). The RM estimates of *F*_inf_ agreed well with those estimated with the sulfur tracer method (*R*^2^ = 0.78; *n* = 14 residences) (Sarnat et al. 2002). We also published estimates of *E*_ag_ and *E*_ig_ for PM_2.5_ among a subset of the Seattle panel study subjects (Allen et al. 2004). We estimated the 24-hr average *E*_ag_ and *E*_ig_ for each subject using the RM *F*_inf_ estimates from the indoor/outdoor nephelometer measurements, the indoor (*C*_i_) and outdoor (*C*_o_) PM_2.5_ concentrations measured with HI_2.5_, and the fraction of the day (*F*_o_) that the subjects reported being outdoors or in transit based on the TAD:


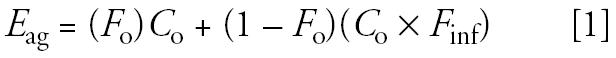



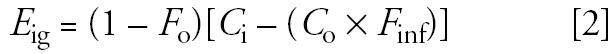


Because nephelometer measurements were only valid at 8 of the 19 subjects’ residences, a predictive model based on RM *F*_inf_ estimates from 62 residences in the Seattle panel study, residence type, outdoor temperature, average daily rainfall, and the use of air cleaners was constructed to estimate *F*_inf_ in the remaining 11 homes (Table 1). The estimated *F*_inf_ values from the predictive model were compared against those from the RM and validated against the conventional sulfur method (Allen et al. 2003), which uses the regression slope of indoor versus outdoor sulfur concentrations for each residence as the estimated *F*_inf_. As a result of calculating *F*_inf_ using both the RM and the predictive model, three groups of *E*_ag_ and *E*_ig_ estimates were created: *a*) those using the RM *F*_inf_ values (*n* = 8 unique subjects), *b*) those using the predictive model *F*_inf_ values (*n* = 11 unique subjects), and *c*) a combination of the above two—that is, RM *F*_inf_ values when available and the predictive model *F*_inf_ for the remaining subjects (henceforth called the combined model; *n* = 8 + 11 = 19 subjects).

### Statistical analysis.

We used a linear mixed effects model with random intercept to test for within-subject associations between eNO and various PM_2.5_ exposure estimates. The model included an interaction term between medication use and PM, a term for the within-subject, within-session (10-day monitoring period) effects, and a term for the subject between-session effects. We adjusted for the confounding variables of temperature, relative humidity, and, in the model for eNO, ambient NO measured at the Beacon Hill site. We also adjusted for subject age and body mass index (BMI). Our primary interest was the within-subject and within-session effect of PM. Analyses were conducted with all children from both winter and spring sessions. STATA 7.0 (Stata Corp., College Station, TX) was used for all health analyses, and SAS statistical package (version 8.0; SAS Institute, Cary, NC) using PROC Genmod with a repeated statement was used for the predictive model *F*_inf_ modeling. All three *E*_ag_/*E*_ig_ data sets (recursive, predictive, and combined) were examined with a focus on the combined data set.

The model used for the eNO analysis was as follows:


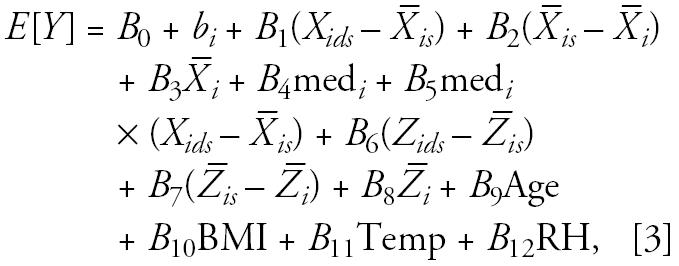


where RH is relative humidity and BMI is body mass index. This basic model was used previously in the original analysis of the relationship between eNO and PM in the children with asthma (Koenig et al. 2003), where *X_ids_* is the PM_2.5_ reading for individual *i* on day *d* during session *s*, X̄*_is_* is the mean PM_2.5_ reading for a subject during a session, X̄*_i_* is the mean PM_2.5_ reading for a subject during one or two sessions, med*_i_* is an indicator for medication use (constant for each subject ), *Z_ids_* is the ambient NO reading for individual *i* on day *d* during session *s*, Z̄*_is_* is the mean ambient NO reading for a subject during a session, and Z̄*_i_* is the mean ambient NO reading for a subject during all sessions.

We also analyzed the data using generalized estimating equations (GEE) with an exchangeable working correlation matrix and robust SEs to adjust for autocorrelation in the data. The GEE model produced similar effect estimates.

## Results

**Figure 1:**
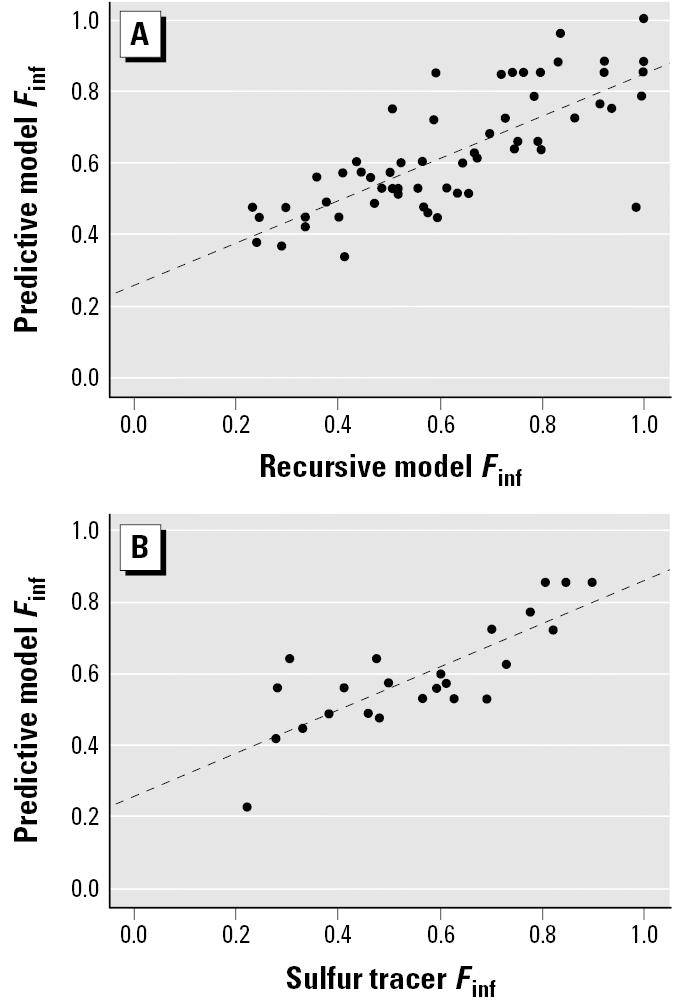
Comparisons between predictive model *F*_inf_ estimates and the *F*_inf_ estimates obtained using the recursive model (*A*; *n* = 62; *y* = 0.59*x* + 0.26; *R*^2^ = 0.60) and the sulfur tracer technique (*B*; *n* = 25; *y* = 0.61*x* + 0.25; *R*^2^ = 0.66).

Nineteen children with asthma participated in this panel study in Seattle. All subjects completed one 10-day monitoring session, and 10 subjects completed two sessions. During this study, the home indoor and outdoor PM_2.5_ concentrations averaged 9.5 and 11.1 μg/m^3^, respectively (Table 2), whereas personal exposure to total PM_2.5_ averaged 13.4 μg/m^3^. The total personal PM_2.5_ exposure was then separated into indoor- and outdoor-originated components using the RM for eight residences with nephelometer measurements and a predictive model for the remaining 11 residences. The predictive model for *F*_inf_ employed two important home characteristics, residence type, and the use of air cleaner, as well as outdoor temperature and precipitation as surrogates for changes of home ventilation conditions (Table 1). This predictive model agreed well with the RM (*R*^2^ = 0.60) and the sulfur tracer *F*_inf_ estimates (*R*^2^ = 0.66) (Figure 1). The average *F*_inf_ for the 19 subjects was 0.56 ± 0.15 (range, 0.23–0.86). The average *E*_ag_ and *E*_ig_ from the RM model were not significantly different from those estimated from the predictive model (Table 2). Thus, we pooled the *E*_ag_ and *E*_ig_ estimates from both models for the following health effect assessment. We examined the *E*_ag_ and *E*_ig_ estimates from the combined model for their associations with increase in eNO. Table 3 shows distributions for the health end points. In this analysis we found that eNO was associated with *E*_ag_ estimated among subjects not on prescribed ICS medication (5.0 ppb per 10-μg/m^3^ increase in estimated exposure; 95% CI, 0.3 to 9.7; Table 4). There was no association between eNO and *E*_ig_ (Table 4). In contrast to our findings with eNO, associations between changes in lung function and estimated exposures were found for *E*_ig_ but not for *E*_ag_. Furthermore, the results were not statistically significant across all lung function measures. FEV_1_ and FVC were both significantly negatively associated with *E*_ig_ in children not using ICS (FEV_1_, *p* = 0.01; FVC, *p* = 0.00), whereas MEF was negatively, but not significantly, associated with *E*_ig_ (*p* = 0.35). No significant associations were seen between lung function changes and the combined model estimates of *E*_ag_.

Table 5 shows associations between the eNO and measured PM_2.5_ on subjects (Harvard personal environmental monitor) and at home indoors and outdoors in the same 19 children included in the combined model. As shown in Table 5, associations were found between eNO and measured outdoor, indoor, and personal PM_2.5_ (*p* = 0.01–0.03). In all cases, the changes were seen only in children not using ICS medications.

## Discussion

Our study has shown that, for eNO, ambient-generated particles are more potent per unit mass than indoor-generated particles. This *E*_ag_ effect on eNO using the combined model estimates also agreed well with the estimates from both the RM and the predictive model. The increases in eNO associated with *E*_ag_ were 5.6 ppb for the RM estimates (*p* = 0.08), 5.3 ppb for the predictive model estimates (*p* = 0.04), and 5.0 ppb for the combined model (*p* = 0.04). Corresponding changes with *E*_ig_ were not significant (*p* = 0.41, 0.12, and 0.15, respectively). In this respect, our results agree with those of Ebelt et al. (in press), who found that outdoor-generated particles were associated with health outcomes, whereas nonambient particles were not in a group of subjects with COPD in Vancouver. These two studies demonstrate the usefulness of separating total personal particle exposures into indoor- and outdoor-generated components and the relative potency of indoor- and outdoor-generated particles.

Our conclusion that eNO is associated more strongly with outdoor-generated particles than indoor-generated particles is supported by the internal consistency of the results. For subjects with combined model estimates of *F*_inf_, the estimated increase in eNO per 10-μg/m^3^ increase in PM_2.5_ was 5.0 ppb (*p* < 0.04) for *E*_ag_, which was greater than the 3.9 ppb for outdoor measured PM_2.5_ (*p* = 0.01) because *E*_ag_ takes into account personal activities and particle infiltration efficiency to arrive at a more accurate estimate of exposure to ambient-originated PM (Table 5). The effect of measured total indoor PM_2.5_, a combination of indoor- and outdoor-generated particles, on eNO was 4.1 ppb/10 μg/m^3^ PM_2.5_ (*p* = 0.01) in Table 5, which was reduced to a nonsignificant 3.3 ppb/10 μg/m^3^ PM_2.5_ (*p* = 0.15) for *E*_ig_ when the ambient PM contribution was removed from the total exposures. In all three exposure models, *E*_ag_ was more strongly associated with eNO than was *E*_ig_. Also, *E*_ag_ showed an interaction with ICS use, as did our original study with outdoor, indoor, and personal measured PM_2.5_ (Koenig et al. 2003).

Our lung function results show that exposure to particles generated indoors, but not outdoors, was associated with decrements of lung functions except for MEF. Furthermore, the association was not consistent across all three exposure models. Both combined (*n* = 17 subjects) and predictive models (*n* = 9 subjects) showed similar results for FEV_1_ and FVC, whereas the recursive model estimates for eight subjects showed nonsignificant association between these lung function measures and *E*_ig_. The fact that some lung function decrements were associated with indoor-generated particles indicates that the relationship between respiratory health and PM is complex. It was not surprising that the PM_2.5_ associations with eNO and lung function were not consistent. This disagreement between eNO increases and lung function changes has been reported in clinical literature that consistently shows either no correlation or a negative correlation between changes in eNO and changes in FEV_1_ among subjects with asthma (Dal Negro et al. 2003; Li et al. 2003; Nightingale et al. 1999; Steerenberg et al. 2003).

Outdoor particle concentrations are associated with a wide spectrum of respiratory health effects including respiratory symptoms in children with asthma (Delfino et al. 1998), lung function decrements in children with asthma (Delfino et al. 2002; Koenig et al. 1993), hospital admissions in the general population (Schwartz 1996; Sheppard et al. 1999), and mortality in the general population (Dockery et al. 1993; Schwartz 2000). On the other hand, there are also studies showing adverse respiratory health effects associated with indoor-generated particles including allergens, dust mites, fungal spores, endotoxins, and viruses (Long et al. 2001; Majid and Kammen 2001; Simoni et al. 2002; Smedbold et al. 2002; Wan and Li 1999).

Our results for eNO appear to be biologically plausible because asthma is an inflammatory disease and perturbations in asthma are expected to be associated with markers of airway inflammation. Several studies show relationships between eNO and outdoor exposure to PM or other air pollutants. One study found an association between exhaled NO values and high levels of outdoor carbon monoxide and NO, but not PM, in the Netherlands in healthy nonsmoking subjects (van Amsterdam et al. 1999, 2000). More recently, eNO levels were associated with exposure to PM_10_, black smoke, nitrogen dioxide, and ambient NO in a panel study of children in the Netherlands (Steerenberg et al. 2001) and in a panel of adults with respiratory disease (Jansen et al. 2004). Adamkiewicz et al. (2004) presented data showing an association between measures of air pollution and eNO values in a panel of elderly nonsmoking subjects with cardiac disease in Steubenville, Ohio (USA). Their analysis found a 1.5-ppb increase in eNO (95% CI, 0.3 to 2.6) for a mean interquartile range increase in PM_2.5_.

### Model limitations.

It is challenging to model personal exposure among children partly because of the elevated personal cloud and children’s movement between several indoor microenvironments (Liu et al. 2003; Wu et al. in press). Children in the Seattle panel study spent an average of 66% of their time indoors at home and 21% indoors away from home (primarily at school), whereas the adults in the larger panel study in Seattle spent an average of 83–88% of their time indoors at home (Liu et al. 2003). Because we only collected stationary indoor measurements and estimated *F*_inf_ in the subjects’ residences, we made a strong assumption that all indoor environments encountered by the subject were represented by their residence. This assumption may have resulted in uncertainties in the exposure estimates because of the considerable fraction of time that this group spent in unmonitored indoor environments, especially school.

To make the most efficient use of our eNO and spirometry data, we developed a predictive model to estimate *F*_inf_ (and therefore *E*_ag_ and *E*_ig_) in residences for which nephelometer data were not available (Table 1). Although the predicted *F*_inf_ estimates were validated with an independent estimate of *F*_inf_ (Figure 1), the predictive model is derived from the estimates produced by the recursive model, and as a result the predictive model estimates include errors introduced by a two-step modeling procedure. Nevertheless, the consistency of the associations between *E*_ag_ and eNO for the RM and the combined model exposure estimates provides evidence of the reliability of the combined model’s *F*_inf_ estimates.

## Conclusion

Our eNO results support our hypothesis that PM_2.5_ of outdoor origin could be more potent per unit mass than particles of indoor origin. However, our lung function data indicate that PM_2.5_ of indoor origin might be more potent per unit mass in resulting in decrements of lung functions, although the results across functional tests were not consistent. If outdoor particles are more strongly associated with adverse health outcomes than particles generated indoors, the fact that outdoor particles readily penetrate indoors would partially explain why epidemiologic time series studies consistently find associations between health outcomes and PM measured at outdoor fixed sites despite the fact that people spend most of their time indoors.

This is a preliminary study using a newly developed exposure source model that we hope will be useful to air pollution epidemiology. We tentatively conclude that partitioning personal exposure into indoor- versus outdoor-generated particles is useful in understanding the health effects of sources of personal PM_2.5_ and that the effects of indoor- versus outdoor-generated particles differ for different health end points.

## Figures and Tables

**Table 1:** Results of regression analysis for *F*_inf_ (*n* = 62 residences).

Parameter	Estimate	SE	95% CI	*p*-Value
Intercept	0.41	0.07	0.28 to 0.54	< 0.001
Residence type				
Private home (reference)				
Private apartment	0.03	0.05	−0.08 to 0.14	0.61
Group home	0.19	0.06	0.07 to 0.31	< 0.01
Air cleaner				
None (reference)				
Ion generator	−0.07	0.05	−0.16 to 0.02	0.14
Filter	−0.08	0.07	−0.22 to 0.05	0.23
Electrostatic precipitator	−0.11	0.06	−0.22 to 0.00	0.05
Average outdoor temperature (°C)*^a^*				
< 4 (reference)				
4–8	0.19	0.07	0.06 to 0.32	< 0.01
8–12	0.32	0.07	0.18 to 0.45	< 0.001
≥12	0.45	0.07	0.31 to 0.58	< 0.001
Average daily rainfall (inches)*^b^*				
< 0.5 (reference)				
0.05–0.1	−0.07	0.05	−0.16 to 0.02	0.13
> 0.1	−0.15	0.06	−0.26 to −0.04	< 0.01

The regression coefficients are used to predict *F*_inf_ in residences without nephelometer data (“predictive model”).

a At Beacon Hill Central Site.

b At Sand Point Way National Weather Service station.

**Table 2:** Distributions of residential indoor and outdoor concentrations and personal *E*_ig_ and *E*_ag_ (μg/m^3^).

Model	Concentration	Total no. of monitoring events*^a^*	No. (days)	Mean	Minimum	25%	Median	75%	Maximum
	Home indoor	27 (19)	248	9.5	2.3	5.7	7.6	10.8	36.3
	Home outdoor			11.1	2.8	6.3	9.5	14.6	40.4
Recursive	*E*_ag_	11 (8)	101	7.0	1.8	4.2	5.9	9.2	22.6
	*E*_ig_			2.1	0.0	0.0	1.2	2.3	17.2
Predictive	*E*_ag_	16 (13)	147	6.0	1.3	3.4	5.0	7.5	22.6
	*E*_ig_			4.0	0.0	0.9	2.2	4.9	33.0
Combined	*E*_ag_	27 (19)	248	6.4	1.3	3.7	5.5	7.8	22.6
	*E*_ig_			3.2	0.0	0.5	1.7	4.2	33.0

Abbreviations: 25%, 25th percentile; 75%, 75th percentile.

a Number of unique subjects in parentheses.

**Table 3:** Descriptive statistics of health outcomes.

Health measurement	No. of subjects (no. sessions)	Person- days	Mean	Minimum	25%	Median	75%	Maximum
eNO (ppb)	19 (29)	240	15.4	5	9.7	12.5	18.0	79.8
FEV_1_ (L)	17 (29)	269	1.8	0.5	1.4	1.9	2.2	3.4
MEF (L/min)	17 (29)	269	113	21	71	107	149	320
FVC (L)	17 (29)	269	2.3	0.7	1.9	2.4	2.7	3.5

Abbreviations: 25%, 25th percentile; 75%, 75th percentile.

**Table 4:** Associations between eNO (ppb) and outdoor- versus indoor-generated particles in children with asthma: recursive model (*n* = 8), predictive model (*n* = 11), and combined model (*n* = 19).

Exposure	Model	Use of medication	Change per 10 μg/m^3^ estimated PM_2.5_	95% CI	*p*-Value
*E*_ig_	Combined	No	3.29	−1.14 to 7.73	0.15
		Yes	−4.94	−10.94 to 1.06	0.11
*E*_ag_	Combined	No	4.98	0.28 to 9.69	0.04
		Yes	1.67	−3.77 to 7.12	0.55
*E*_ig_	Recursive	No	−0.19	−8.37 to 8.00	0.97
		Yes	−0.47	12.03 to 11.10	0.94
*E*_ag_	Recursive	No	5.63	−0.62 to 11.88	0.08
		Yes	−4.30	−14.60 to 6.01	0.41
*E*_ig_	Predictive	No	3.46	−0.90 to 7.83	0.12
		Yes	−4.99	−11.01 to 1.04	0.11
*E*_ag_	Predictive	No	5.33	0.31 to 10.35	0.04
		Yes	1.66	−3.75 to 7.06	0.55

**Table 5:** Results of eNO analyses with indoor, outdoor, and personal monitors for 19 children included in the combined model.

Measure	Use of medication	Change per 10 μg/m^3^ estimated PM_2.5_	95% CI	*p*-Value
Personal*^a^*	No	4.48	0.95 to 8.00	0.01
	Yes	−0.49	−2.95 to 1.98	0.70
Outdoor	No	3.90	0.91 to 6.88	0.01
	Yes	1.00	−2.10 to 4.09	0.53
Indoor	No	4.13	0.87 to 7.38	0.01
	Yes	−1.37	−5.44 to 2.70	0.51

a Two sessions removed from personal PM analysis because of insufficient data.
